# Indoor Dust as a Matrix for Surveillance of COVID-19

**DOI:** 10.1128/mSystems.01350-20

**Published:** 2021-04-13

**Authors:** Nicole Renninger, Nicholas Nastasi, Ashleigh Bope, Samuel J. Cochran, Sarah R. Haines, Neeraja Balasubrahmaniam, Katelyn Stuart, Aaron Bivins, Kyle Bibby, Natalie M. Hull, Karen C. Dannemiller

**Affiliations:** a Department of Civil, Environmental & Geodetic Engineering, College of Engineering, Ohio State University, Columbus, Ohio, USA; b Environmental Sciences Graduate Program, Ohio State University, Columbus, Ohio, USA; c Division of Environmental Health Sciences, College of Public Health, Ohio State University, Columbus, Ohio, USA; d Sustainability Institute, Ohio State University, Columbus, Ohio, USA; e Department of Civil & Environmental Engineering & Earth Sciences, College of Engineering, University of Notre Dame, Notre Dame, Indiana, USA; University of Illinois at Urbana-Champaign

**Keywords:** SARS-CoV-2, COVID-19, dust, surveillance, virus, indoor environment, built environment, vacuum, outbreak, monitoring

## Abstract

Ongoing disease surveillance is a critical tool to mitigate viral outbreaks, especially during a pandemic. Environmental monitoring has significant promise even following widespread vaccination among high-risk populations. The goal of this work is to demonstrate molecular severe acute respiratory syndrome coronavirus 2 (SARS-CoV-2) monitoring in bulk floor dust and related samples as a proof of concept of a noninvasive environmental surveillance methodology for coronavirus disease 2019 (COVID-19) and potentially other viral diseases. Surface swab, passive sampler, and bulk floor dust samples were collected from the rooms of individuals positive for COVID-19, and SARS-CoV-2 was measured with quantitative reverse transcription-PCR (RT-qPCR) and two digital PCR (dPCR) methods. Bulk dust samples had a geometric mean concentration of 163 copies/mg of dust and ranged from nondetects to 23,049 copies/mg of dust detected using droplet digital PCR (ddPCR). An average of 89% of bulk dust samples were positive for the virus by the detection methods compared to 55% of surface swabs and fewer on the passive sampler (19% carpet, 29% polystyrene). In bulk dust, SARS-CoV-2 was detected in 76%, 93%, and 97% of samples measured by qPCR, chip-based dPCR, and droplet dPCR, respectively. Detectable viral RNA in the bulk vacuum bags did not measurably decay over 4 weeks, despite the application of a disinfectant before room cleaning. Future monitoring efforts should further evaluate RNA persistence and heterogeneity in dust. This study did not measure virus infectivity in dust or potential transmission associated with dust. Overall, this work demonstrates that bulk floor dust is a potentially useful matrix for long-term monitoring of viral disease in high-risk populations and buildings.

**IMPORTANCE** Environmental surveillance to assess pathogen presence within a community is proving to be a critical tool to protect public health, and it is especially relevant during the ongoing COVID-19 pandemic. Importantly, environmental surveillance tools also allow for the detection of asymptomatic disease carriers and for routine monitoring of a large number of people as has been shown for SARS-CoV-2 wastewater monitoring. However, additional monitoring techniques are needed to screen for outbreaks in high-risk settings such as congregate care facilities. Here, we demonstrate that SARS-CoV-2 can be detected in bulk floor dust collected from rooms housing infected individuals. This analysis suggests that dust may be a useful and efficient matrix for routine surveillance of viral disease.

## OBSERVATION

The spread of the novel severe acute respiratory syndrome coronavirus 2 (SARS-CoV-2) reached pandemic designation in March 2020 and has since resulted in more than 75 million cases of coronavirus disease 2019 (COVID-19) and 1.6 million deaths documented worldwide as of 21 December 2020 [WHO Coronavirus Disease (COVID-19) Dashboard (https://covid19.who.int)]. Both symptomatic and asymptomatic carriers shed the virus into the environment (1–3). Viral particles are shed primarily via respiratory droplets and aerosols and persist on surfaces indoors (3–5). SARS-CoV-2 persistence has been characterized after deposition onto several surface types and under different environmental conditions (5, 6). In one study, infectious virus was detected on plastics and stainless steel up to 72 h after application (4). Other studies have demonstrated respiratory viruses can contaminate environmental dust near infected individuals (7–9). These viral shedding routes together with persistence indoors and in environmental dust implicate potential viral contamination of indoor dust near infected individuals (10).

There is a critical need for targeted, efficient, and inexpensive methods to monitor SARS-CoV-2 and other viruses in the long term to identify potential viral outbreaks prior to extensive spread. Fecal shedding of SARS-CoV-2 provides the basis for large-scale viral monitoring in wastewater systems (11–14). However, more targeted monitoring efforts are needed for indoor environments, especially those housing vulnerable populations such as congregate care facilities. We propose that the detection of SARS-CoV-2 RNA in indoor dust can be used for continued environmental surveillance of the novel coronavirus, SARS-CoV-2. Targeted monitoring of dust in high-concern buildings could complement broader population-level monitoring approaches. This strategy could then be extended to other viruses of concern. Our goal is to demonstrate that indoor dust can be used as a matrix for viral surveillance.

### Findings.

**FIG 1:**
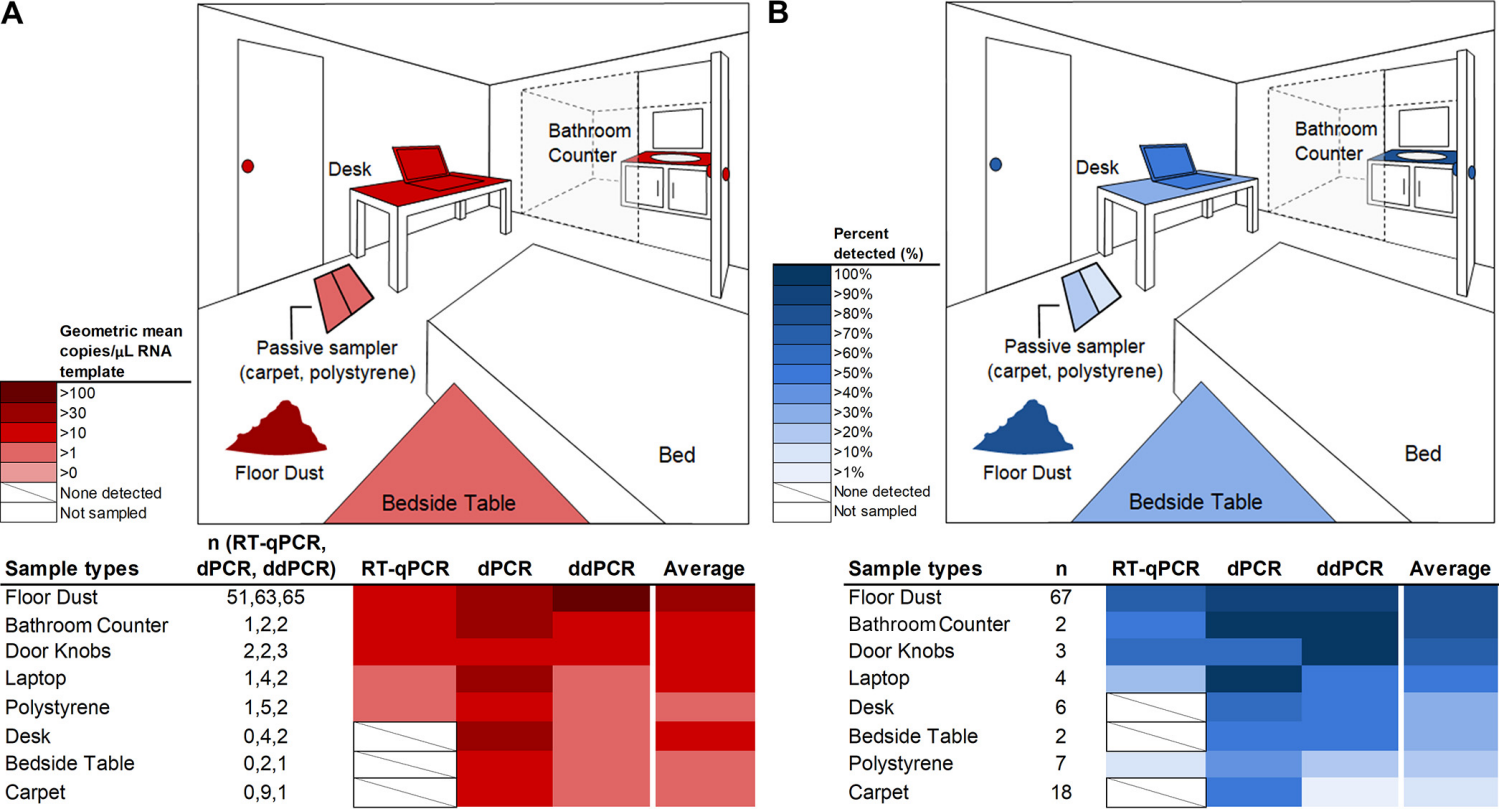
Heatmap displaying the geometric mean number of copies per microliter of RNA template of samples above the detection limit (samples below detection limit were excluded) (A) and the proportion of samples positive (as a percentage) (B) for bulk dust, surface swabs, and passive sampler as observed with three PCR-based methods. The variable n is the number of samples used to calculate the geometric mean (positive detects) in panel A or proportion of samples positive in panel B. For “Floor Dust,” n refers to the total number of bulk dust samples tested for SARS-COV-2, including the dust samples tested weekly over a period of 4 weeks. Items in white on the room diagram were not sampled, and items in white with a slash in the lower heatmaps were not detected. Colors on the room diagrams represent the average value among all three measurement methods.

We measured SARS-CoV-2 using quantitative reverse transcription PCR (RT-qPCR), chip-based digital PCR (dPCR), and droplet digital PCR (ddPCR) in samples of bulk dust, passive surface samples, and surface swabs from rooms of individuals with COVID-19. In bulk dust, the SARS-CoV-2 viral concentration had a geometric mean value of 163 copies/mg of dust and ranged from nondetects to 23,049 copies/mg of dust (Fig. 1A). We detected SARS-CoV-2 RNA in 89% of bulk dust, 55% of surface swabs, and 21% of passive surface sampler samples (average among all three detection methods used, Kruskal-Wallis *P* = 0.02). The ddPCR method detected viral RNA in 97% of bulk dust samples compared to 93% for the chip-based dPCR and 76% for RT-qPCR (Kruskal-Wallis *P* = 0.37) (Fig. 1B). Across all sample types, the ddPCR method detected viral RNA in 60% of samples compared to 71% for the chip-based dPCR and 29% for RT-qPCR (Kruskal-Wallis *P* = 0.06).

**FIG 2:**
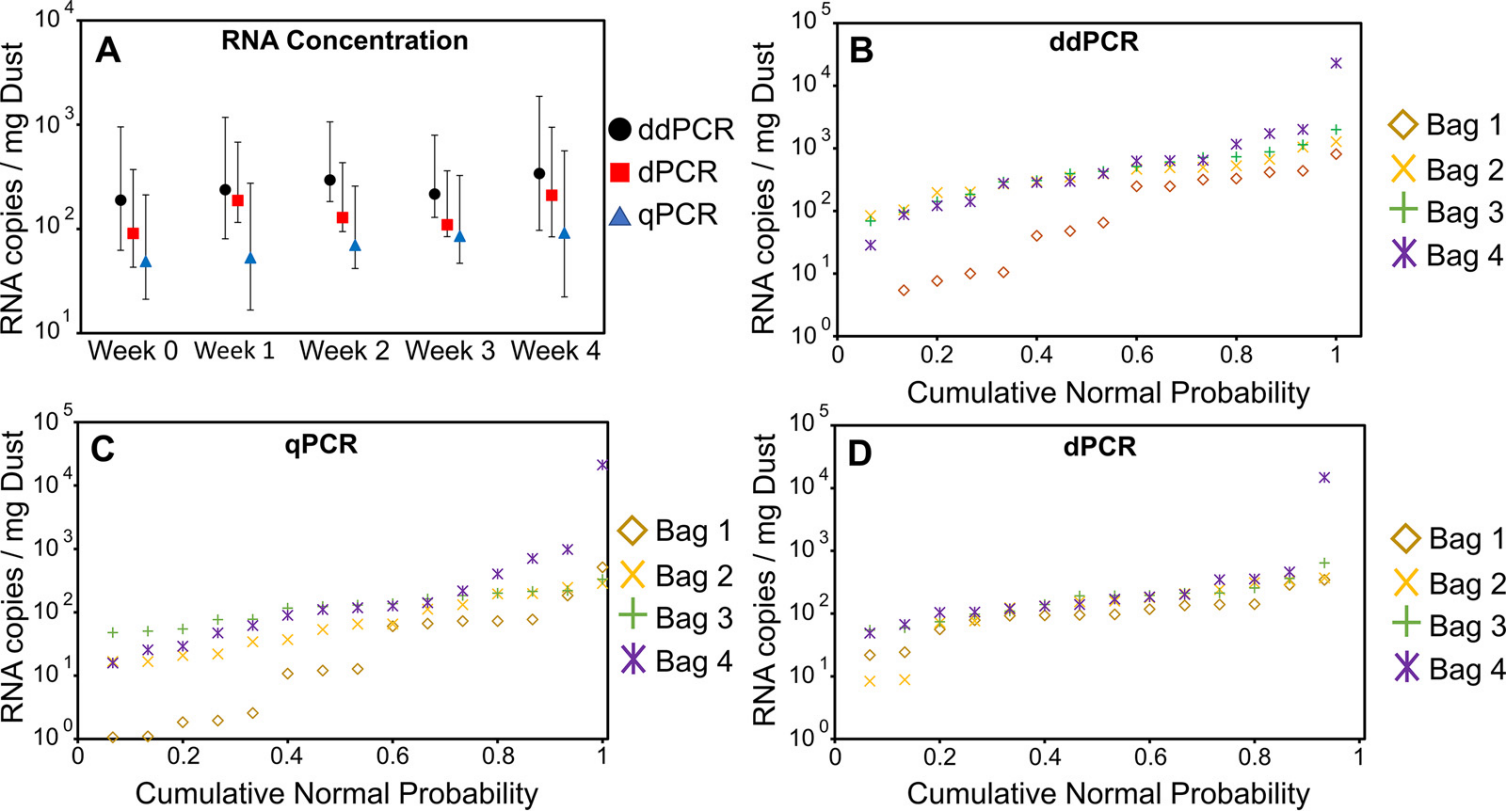
(A) RNA concentration of bulk dust samples (average of four bags) from initial collection to 4 weeks as measured by ddPCR, dPCR, and qPCR. Error bars shown represent the 95% confidence interval of each measurement. (B to D) Cumulative normal probability plots for each measurement method show variability of RNA concentration values for each bulk bag collected.

The COVID-19 isolation rooms were treated with a chlorine-based disinfectant prior to dust collection as part of the normal cleaning process, and the disinfectant is expected to largely inactivate the virus through reactions with the viral capsid (15). The bags were stored in the laboratory at room temperature after collection. Triplicate subsamples were extracted, and viral RNA was measured immediately upon collection and once per week for 4 weeks. Viral RNA did not measurably decay over 4 weeks in the vacuum bags (regression *R*^2^ = 0.009, *P* = 0.47) (Fig. 2A). The coefficient of variance (CV) for number of copies/mg of dust ranged from 73.5 to 313.4% within each vacuum bag when averaged across the three methods of viral detection. This large variation in viral concentration is likely due to the heterogeneous mixture in the bags (Fig. 2B to D).

### Discussion.

The novel coronavirus and the ongoing COVID-19 pandemic have highlighted the need for sensitive and scalable viral surveillance within communities. In the long term, the threat of COVID-19 outbreaks will subside to a level where indefinite routine testing of asymptomatic individuals may be too cumbersome or expensive. However, there will continue to be a need to more broadly monitor vulnerable populations such as those in long-term-care facilities or high-risk patients in hospitals for SARS-CoV-2, influenza, respiratory syncytial virus (RSV), and other emerging viral diseases. Novel pathogens can be targeted with adaptable PCR-based assays. After detection, outbreaks can then be addressed with more targeted resources such as direct patient testing.

Our results demonstrate that environmental dust collection may provide a convenient and useful matrix for ongoing viral monitoring. The process can provide monitoring for many high-risk individuals, and dust samples are already being collected through normal cleaning practices such as vacuuming. Dust had a higher positivity rate than surface swab samples, and the positivity rate of the surface swabs in this study was similar to or greater than the rates in similar studies (16, 17). Our observations indicate that SARS-CoV-2 RNA in dust can persist at least 4 weeks after dust collection and that the measured concentration can vary in different dust subsamples within a vacuum bag. Therefore, multiple samples should be taken from a bag to more rigorously quantify the viral genetic signal, or homogenization methods should be developed that comply with biosafety standards. Additionally, RNA and dust persistence in the environment should be considered when determining if the outbreak occurred recently or in the past. Differences between PCR-based measurement methods may inform method choice. For instance, RT-qPCR requires calibration standards for quantification and the digital methods do not, and for the assays used, ddPCR is a one-step reaction and the chip-based dPCR requires a two-step reaction. Each instrument also has a different detection limit and resulted in marginally different positivity rates. Previously, measurements of indoor environmental microbes have been used to detect infectious microbes such as Aspergillus fumigatus and Legionella pneumophila (18–20). However, nucleotide-based tests do not measure infectivity, meaning the detection of genetic material from these microbes may indicate that people in the area are infected but would not necessarily indicate the risk of infection due to contact with indoor surfaces or via resuspension of floor dust.

Indoor dust may also be used to complement other environmental surveillance methods, e.g., wastewater monitoring. Wastewater detection may be more beneficial at larger population scales covering thousands of individuals in a community, and one infected individual may be detected among 100 to 2,000,000 individuals (21). Indoor dust may be useful in areas with smaller numbers of high-risk individuals where more specific outbreak identification is critical. Additionally, not all individuals secrete virus in stool (22). Indoor dust sampling may also be less expensive and be easier to implement, with simplified sample collection and no preconcentration steps of samples required. Other dust collection methods are available beyond those described in this study. Future research should evaluate differences between collection strategies.

Limitations of this study include that we did not measure the infectivity of SARS-CoV-2 in the dust samples due to biosafety constraints, although this is not needed for surveillance. Also, our small sample size from rooms occupied by infected students may not be representative of other buildings and occupancy conditions, and samples were collected after a known infection as opposed to before. More information is needed on how representative each dust sample would be for a specific population and different occupancy levels. We were unable to sieve or otherwise homogenize the dust due to biosafety concerns, which likely resulted in variability within vacuum bags (Fig. 2B to D). Additionally, decay of viral RNA in dust on a floor may differ from decay of viral RNA in dust treated with disinfectant and stored in a vacuum bag.

### Conclusions.

Indoor dust provides an important matrix for environmental surveillance of viral disease outbreaks. Infected humans shed virus into their surrounding environment, which becomes integrated into the dust. In many cases, dust is already being collected during routine cleaning and can easily be submitted for analysis. Overall, dust may be a useful and efficient matrix to provide identification of viral disease in high-risk settings, such as congregate care facilities. Future research can validate these results on a broader scale and in different building types to better inform use of this technique to mitigate viral transmission.

### Experimental protocol. (i) Overview.

Samples were collected from two different homes, as well as isolation rooms used to quarantine individuals who tested positive for SARS-COV-2. Bulk dust was collected from both homes, and four composite samples were collected from 30 to 50 student isolation rooms each. Surface swabs and a passive sampler collection were completed in one home. Viral RNA was measured using RT-qPCR, chip-based digital PCR, and droplet digital PCR.

### (ii) House surface swabs and passive sampler collection.

Surface and passive samples were collected at the end of the 10-day quarantine period from two bedrooms of individuals who tested positive for SARS-CoV-2 in house 1. Surfaces were swabbed using sterile flocked swabs (Puritan, ME, USA), and passive samplers consisting of carpet coupons and polystyrene coupons were placed on the floors of their isolation rooms. The passive samplers consisted of three cut pile carpet squares (fiber length, 10 mm), three loop pile carpet squares (fiber length, 7.5 mm), and three polystyrene squares attached to a template. All squares were 5 cm × 5 cm each. Both carpet types used were made of 100% polyethylene terephthalate (PET) fibers and a synthetic jute backing material. Fibers were specifically manufactured to contain no antimicrobial, stain, or soil resistance coatings. Swabs were dipped in autoclaved phosphate-buffered saline (PBS), and each was used on a different 10 cm × 10 cm surface area. Each wetted swab was wiped left and right across the 10 cm × 10 cm surface area, rotated 1/3 turn, and wiped to cover the surface area up and down, rotated a final 1/3 turn, and wiped in circular motions across the surface area. Swabs were placed back in the corresponding tube and resealed until extraction.

In room 1, two swabs were used for the desk, two for a bedside table, and two for a computer. A passive sampler was placed on the floor by the bed for 4 days. In room 2, two swabs were used for 100-cm^2^ areas on a computer, two for the same areas on a desk, two for the same areas on a second desk, one for the doorknob of the bedroom, two for 100-cm^2^ areas on the bathroom counter, and two on the bathroom doorknob, one for each side of the door. A passive sampler was placed on the floor between the desk and bed for 2 days, and another was placed on the open space in the bedroom for 4 days.

### (iii) House bulk dust.

Bulk floor dust was collected from occupant vacuum bags of two different houses that had individuals infected with COVID-19. House 1 had floor dust collected 27 days after quarantine ended. House 2 had floor dust collected in the middle of the quarantine period.

### (iv) Isolation room bulk dust.

Bulk dust samples were retrieved from four different vacuum bags used to clean the isolation rooms for students with COVID-19 at Ohio State University in Columbus, OH, USA, and extracted over 4 weeks. Vacuum bags were collected by cleaning staff from rooms that were used to house students who tested positive for SARS-COV-2. One or two students would isolate in the rooms for 10 days after a positive diagnosis. The cleaning staff would vacuum and clean the rooms after the quarantine period was over and within 18 h of the students leaving the rooms. Cleaning staff would spray the room with an electrostatic sprayer containing a disinfectant (sodium dichloro-s-triazinetrione, CAS 2893-78-9) and wait at least 20 min prior to vacuuming. This disinfectant provides free chlorine (stabilized by cyanuric acid), which nonselectively oxidizes biomolecules to inactivate pathogens. It is possible the disinfectant may be depleted by reacting with other organic material and biomolecules (dead skin, etc.) and viral capsids in dust samples before impacting viral RNA (15). Cleaning staff used a Windsor Sensor XP12 vacuum (Kärcher, Denver, CO) to collect dust over a 3- to 4-week period. Each vacuum bag contained dust from approximately 30 to 50 isolation rooms as well as hallways, and potentially from surfaces in the isolation rooms if considered dusty. The isolation room flooring was vinyl composite tile, and the hallway flooring was wall to wall carpet.

### (v) RNA extraction.

Viral RNA was extracted from dust and surface samples. Bulk dust samples and surface swabs were extracted using a Qiagen RNeasy Powermicrobiome extraction kit procedure (Qiagen, Hilden, Germany) modified to include 10 times the recommended concentration of 2-mercaptoethanol and using phenol-based lysis. Triplicates of approximately 50 mg of dust were removed from bulk dust samples using an autoclaved spatula, and each replicate was extracted individually in a laminar flow biosafety cabinet. The spatula was flame sterilized between removing replicates. Isolation room bulk dust was extracted over a period of 4 weeks in which triplicate dust samples were extracted from the same bag after initial collection and then again each week for 4 weeks for a total of 60 samples of this type. Bulk dust from student isolation rooms was stored in sealed bags and kept at a room temperature of approximately 22.8°C with a room relative humidity that fluctuated from 15 to 30%. Dust was not sieved due to biosafety concerns. Swabs were placed directly into the lysis tubes for extraction. Carpet samples from the passive sampler were extracted using the QIAmp DSP Viral RNA minikit (Qiagen, Hilden, Germany). A 3 cm × 1 cm area was cut out of the middle of each carpet to reduce potential edge effects and vortexed for 1 min in 4,000 μl of autoclaved PBS. A total of 140 μl of this wash liquid was used in the RNA extraction. Swabs were dipped in autoclaved PBS and wiped horizontally and vertically across the polystyrene pieces on the passive sampler. All extraction sets included a blank to detect potential contamination. The RNA extract of a prepandemic dust sample collected in September 2019 was also tested and shown to be negative for SARS-CoV-2 on RT-qPCR with no amplification.

### (vi) Viral detection. *(a)* RT-qPCR.

The viral detection assay targeted the N1 gene using the IDT SARS-CoV-2 (2019-nCoV) CDC qPCR probe assay (Integrated DNA Technologies, Inc., Coralville, IA, USA). This assay uses the 2019-nCoV_N1 forward primer (GAC CCC AAA ATC AGC GAA AT), the 2019-nCov_N1 reverse primer (TCT GGT TAC TGC CAG TTG AAT CTG), and the 2019-nCoV_N1 probe (6-carboxyfluorescein [FAM]-ACC CCG CAT/ZEN/TAC GTT TGG ACC-3' Iowa Black FQ [3IABkFQ]). Direct one-step real-time qPCR amplification of cDNA was performed using qScript XLT one-step RT-qPCR ToughMix (Quanta BioSciences, Gaithersburg, MD, USA). Each well contained 5 μl of RNA template, 10 μl of qScript XLT one-step RT-qPCR ToughMix, 1.5 μl of the IDT SARS-CoV-2 forward and reverse primers at 500 nM and probe at 125 nM, and 3.5 μl of sterile deionized (DI) water. The 2019-nCoV plasmid control 10-fold serial dilutions were used as a standard curve to calculate the number of copies per microliter of RNA template, based on plasmid quantification determined by dPCR (see below) (Integrated DNA Technologies, Inc., Coralville, IA, USA). Cycling parameters were set following the instructions supplied by the CDC for qScript XLT one-step RT-qPCR ToughMix (23). Cycling parameters consisted of 10 min at 50°C for 1 cycle, 3 min at 95°C for 1 cycle, and 3 s at 95°C followed by 30 s at 55°C for 50 cycles. Seven no-template controls were tested, and no amplification occurred.

A subset of 10% of samples were tested for inhibition. The RNA template was spiked with positive plasmid control to test for a reduction in signal due to the presence of inhibitors. The spike concentration was 100 times the highest sample concentration determined by qPCR. Inhibition was indicated if there was a delay in expected amplification. Each sample type was tested for inhibition: bulk dust, swab, and passive sampler. No inhibition was detected in any of the sample types except for the carpet wash from the passive sampler, where the inhibition delayed amplification by 1.45 cycles. Diluting these samples by 10-fold to reduce inhibition would place these samples below the detection limit of 2.3 copies per μl of RNA.

### *(b)* Chip-based dPCR.

Digital PCR was performed using the QuantStudio 3D Digital (QS3D) PCR system (Applied Biosystems, Forest City, CA) that utilizes a chip-based technology. This system uses a QuantStudio 3D Digital PCR Chip Adapter kit for the ProFlex Flat Block thermal cycler equipped with a tilt base, which holds the chips (version 2) in place during thermocycling. cDNA was first reverse transcribed from RNA samples using the iScript cDNA Synthesis kit (Bio-Rad, Hercules, CA) according to the recommended reaction protocol on the ProFlex PCR system (Applied Biosystems, Forest City, CA). RNA was detected and quantified using the N1 assay described above. Each reaction was prepared as a 15-μl volume consisting of 2.00 μl of water, 7.25 μl of QuantStudio 3D Digital PCR Master Mix v2 (Applied Biosystems, Forest City, CA), forward and reverse primers at 500 nM, probe at 125 nM, and 5 μl of RNA extract. A portion (14.5 μl) of the solution was transferred into the sample loading port of the loading blade and then loaded onto the chip. Immersion fluid was used to cover the surface of the chip. The chip was then sealed, and additional immersion fluid was added to fill the chip case. Thermal cycling consisted of 10 min at 96°C, 39 cycles of 60°C for 2 min followed by 98°C for 30 s, and finally 60°C for 2 min. The cover temperature was set at 70°C, and the reaction volume was set at 1 nl. Each experiment included one negative control and one N1 positive control (2019-nCoV_N_Positive Control; Integrated DNA Technologies, Inc., Coralville, IA, USA). Chips were then removed and imaged using the QuantStudio 3D digital PCR instrument following thermal cycling. Manual thresholding and quantification were performed using the QuantStudio 3D AnalysisSuit Software. The 95% limit of blank for the N1 assay was determined to be 1.45 gene copies per μl of reaction mixture using seven replicates of negative controls. Inhibition was not assessed for dPCR.

### *(c)* ddPCR.

Droplet digital PCR was performed using the Bio-Rad QX200 system along with a C1000 Touch thermal cycler (Bio-Rad, Hercules, CA). SARS-CoV-2 RNA was detected and quantified using the N1 assay previously described. Inhibition was assessed by spiking a subset of sample extracts (*n* = 17) with bovine respiratory syncytial virus (BRSV) RNA extracted directly from a live attenuated bovine vaccine (Inforce 3 cattle vaccine; Zoetis, Parsippany-Troy Hills, NJ) using a Qiagen PowerViral AllPrep DNA/RNA kit (Hilden, Germany). BRSV RNA was detected and quantified using an assay targeting the nucleoprotein gene with forward primer (GCA ATG CTG CAG GAC TAG GTA TAA T), reverse primer (ACA CTG TAA TTG ATG ACC CCA TTC T), and probe (FAM-ACC AAG ACT/ZEN/TGT ATG ATG CTG CCA AAG CA-3IABkFQ) (Integrated DNA Technologies, Inc., Coralville, IA, USA) (24). Each N1 ddPCR reaction mixture was prepared as a 22-μl volume consisting of 5.45 μl of water, 5.45 μl of one-step RT-ddPCR Supermix (Bio-Rad, Hercules, CA), 2.1 μl of reverse transcriptase, 1.05 μl of dithiothreitol, forward and reverse primers at 1,000 nM, probe at 250 nM, and 5 μl of RNA extract. BRSV wells were prepared in the same manner except that forward and reverse primers at 900 nM and probe at 250 nM were used. A volume of 20 μl of each reaction mixture was passed into droplet generation. Thermal cycling was performed with reverse transcription for 60 min at 50°C, followed by 10 min at 95°C, 40 cycles of 95°C for 30 s followed by 59°C for 1 min, and finally 98°C for 10 min. Each ddPCR experiment included two no-template controls each for BRSV and N1 and two positive controls each for BRSV (RNA and molecular water) and N1 (2019-nCoV_N_Positive control; Integrated DNA Technologies, Inc., Coralville, IA, USA). Manual thresholding and quantification were performed in QuantaSoft version 1.7.4 (Bio-Rad, Hercules, CA) so that no-template controls yielded no positive droplets.

The 95% limit of detection for the N1 assay was determined to be 3.3 gene copies per ddPCR reaction using a 10-replicate dilution series of synthetic SARS-CoV-2 RNA control material (catalog no. MT188340; Twist Bioscience, San Francisco, CA) with a cumulative Gaussian distribution fit to the observed proportion of the replicates positive along the dilution gradient. There was no evidence of inhibition as no difference was observed in the quantification of BRSV RNA in sample extracts compared to the BRSV positive controls (two-tailed *t* test, *P* = 0.19).

### Statistical and data analyses.

Our goal was to compare measurement of SARS-CoV-2 in bulk dust, on surface swabs, and on a passive sampler using three different measurement methods. Each vacuum bag of dust was sampled and extracted in triplicate at each time point (immediately after collection and 1, 2, 3, and 4 weeks after collection). All three detection methods (qPCR, dPCR, and ddPCR) analyzed the same sample extractions for all sample types. Differences in positivity rates among detection methods and sample types were assessed using the Kruskal-Wallis H test. Detection limit information is described above for each detection method. The geometric mean was reported for quantification of SARS-CoV-2 RNA present in samples using each method due to the logarithmic nature of PCR-based data. Potential RNA decay over the 4-week time period was evaluated in bulk dust with a regression analysis on the ddPCR data transformed with the natural logarithm. The data set is available at https://doi.org/10.5061/dryad.3n5tb2rg1.
